# Long COVID Discourse in Canada, the United States, and Europe: Topic Modeling and Sentiment Analysis of Twitter Data

**DOI:** 10.2196/59425

**Published:** 2024-12-09

**Authors:** Ahmed Ghassan Tawfiq AbuRaed, Emil Azuma Prikryl, Giuseppe Carenini, Naveed Zafar Janjua

**Affiliations:** 1 Department of Computer Science The University of British Colombia Vancouver, BC Canada; 2 NOSM University Thunder Bay, BC Canada; 3 BC Centre for Disease Control Vancouver, BC Canada

**Keywords:** long COVID, topic modeling, sentiment analysis, Twitter, public perception, social media analysis, public health

## Abstract

**Background:**

Social media serves as a vast repository of data, offering insights into public perceptions and emotions surrounding significant societal issues. Amid the COVID-19 pandemic, long COVID (formally known as post–COVID-19 condition) has emerged as a chronic health condition, profoundly impacting numerous lives and livelihoods. Given the dynamic nature of long COVID and our evolving understanding of it, effectively capturing people’s sentiments and perceptions through social media becomes increasingly crucial. By harnessing the wealth of data available on social platforms, we can better track the evolving narrative surrounding long COVID and the collective efforts to address this pressing issue.

**Objective:**

This study aimed to investigate people’s perceptions and sentiments around long COVID in Canada, the United States, and Europe, by analyzing English-language tweets from these regions using advanced topic modeling and sentiment analysis techniques. Understanding regional differences in public discourse can inform tailored public health strategies.

**Methods:**

We analyzed long COVID–related tweets from 2021. Contextualized topic modeling was used to capture word meanings in context, providing coherent and semantically meaningful topics. Sentiment analysis was conducted in a zero-shot manner using Llama 2, a large language model, to classify tweets into positive, negative, or neutral sentiments. The results were interpreted in collaboration with public health experts, comparing the timelines of topics discussed across the 3 regions. This dual approach enabled a comprehensive understanding of the public discourse surrounding long COVID. We used metrics such as *normalized pointwise mutual information* for coherence and *topic diversity* for diversity to ensure robust topic modeling results.

**Results:**

Topic modeling identified five main topics: (1) long COVID in people including children in the context of vaccination, (2) duration and suffering associated with long COVID, (3) persistent symptoms of long COVID, (4) the need for research on long COVID treatment, and (5) measuring long COVID symptoms. Significant concern was noted across all regions about the duration and suffering associated with long COVID, along with consistent discussions on persistent symptoms and calls for more research and better treatments. In particular, the topic of persistent symptoms was highly prevalent, reflecting ongoing challenges faced by individuals with long COVID. Sentiment analysis showed a mix of positive and negative sentiments, fluctuating with significant events and news related to long COVID.

**Conclusions:**

Our study combines natural language processing techniques, including contextualized topic modeling and sentiment analysis, along with domain expert input, to provide detailed insights into public health monitoring and intervention. These findings highlight the importance of tracking public discourse on long COVID to inform public health strategies, address misinformation, and provide support to affected individuals. The use of social media analysis in understanding public health issues is underscored, emphasizing the role of emerging technologies in enhancing public health responses.

## Introduction

As of December 2023, there have been more than 700 million cases of COVID-19 globally, leading to nearly 7 million deaths [1]. These figures likely constitute an underestimation, given reduced reporting requirements for COVID-19 in most countries.

Beyond the acute effects of infection, a significant portion of survivors of COVID-19 experience a broad spectrum of ongoing symptoms several months after infection, which are generally captured under terms such as long COVID and post–COVID-19 condition. The estimated proportion of survivors with long COVID varies extremely widely from below 10% to around 60% across different studies [2], but population-based studies have reported that about 20% of people developed long COVID [3].

While the definition of long COVID continues to evolve, it has been defined as new, returning, or ongoing health problems that persist or occur 4 or more weeks after SARS-CoV-2 infection, with wide-ranging symptoms that can include fatigue, postexertional malaise, shortness of breath, palpitations, trouble sleeping, cognitive deficits, anxiety, and depression [4].

The characteristics and subtypes of long COVID, which likely represents several overlapping entities, continue to be an active area of research using both traditional observational study designs [5] and other innovative data-driven approaches [6].

During the COVID-19 pandemic, people have used social media such as Twitter (rebranded as “X” in 2023) to share information, opinions, and sentiments about COVID-19 and long COVID. This kind of information can help inform health care organizations and public health organizations and assist them in developing approaches and interventions that are sensitive to the concerns and perceptions of the public. This is of relevance to long COVID because it is a diverse condition that is experienced and perceived by the public in many different ways, making the identification of key issues and concerns associated with the condition of interest to health organizations to inform their communications, interventions, and research objectives around long COVID.

The use of topic modeling and sentiment analysis is widespread for identifying issues and public opinions in the field of public health. These approaches are also being used to gain insights into COVID-19–related matters. Analyses have been performed to unveil patterns in health communications across a variety of data sources, communities, and geographic locations.

While certain studies explored news articles [7] or research papers [8,9], the majority of research concentrated on social media platforms such as Reddit posts [10,11] and tweets [10,12-31]. Various approaches have been used to address the tasks of topic modeling, its visualization, and sentiment analysis.

Latent Dirichlet allocation (LDA) has been widely regarded as the best approach for topic modeling until recent years, due to its probabilistic foundation and effectiveness in uncovering latent themes within a corpus of text [32]. While most of the studies have used LDA to achieve topic modeling [8,12,14,16,20,24,33,34], Ridhwan and Hargreaves [13] have used Gibbs Sampling Dirichlet Multinomial Mixture [35], while Zheng et al [36] used conditional random field for a named entity recognition task aiming to extract the most frequent terms and applying the Jaccard similarity coefficient. Furthermore, Yan et al [37] applied biterm topic modeling, and Sussman et al [10] have used Cision’s Brandwatch software to perform both topic modeling and sentiment analysis.

On the other hand, sentiment analysis has been tackled by various rule-based and machine learning methods. Valence Aware Dictionary for Sentiment Reasoning [38] is a famous rule-based system that has been used by many studies [12-14]. TextBlob is a library for processing text that also implements a rule-based sentiment analysis system used by several studies [17,20,22]. Marcec and Likic [18] have used the AFINN lexicon [39], a list of English terms manually rated for valence.

Moreover, other studies have used machine learning methods to improve sentiment analysis. These include classical machine learning methods such as logistic regression, AdaBoost [39], XGBoost [40], and advanced techniques such as long short-term memory networks [41], which have been used by Guo et al [21]. Multilayer perceptron [42], naïve Bayes [43], and support vector machine [44] algorithms have been used by Masood et al [23]. In addition, Kumar et al [19] used bidirectional encoder representations from transformers [45], a deep neural network based on transformer architecture, demonstrating the efficacy of these advanced models in capturing the nuances of sentiment in social media text.

By integrating these approaches, researchers have been able to develop more sophisticated models for sentiment analysis, contributing to a deeper understanding of public opinion and emotional responses on social media platforms. Moreover, some studies have also explored sentiments in the context of specific aspects selected by domain experts [46]. However, such methods have not been used extensively to investigate issues related to long COVID, except for a few select papers (Table 1). While the research involved variations of topic modeling or sentiment analysis techniques, they generally did not combine these approaches.

**Table 1:** Related work on topic modeling and sentiment analysis on long COVID–related data^a^.

Authors	Source	Posters	Time	Location	Language	Topic modeling	Sentiment analysis
Déguilhem et al [47]	Twitter, Reddit, Doctissimo, Facebook, and other forums	Public	January 1, 2020, to August 10, 2021	France	French	Yes, biterm topic modeling [37]	No
Bhattacharyya et al [48]	Twitter	Public	August 28, 2022, to September 6, 2022	Not specified	English	No	Yes, National Research Council Emotion Lexicon [49]
Southwick et al [33]	Reddit	Public	Not specified	Not specified	English	Yes, LDA^b^ [32]	Yes, Affective Norms for English Words lexicon [50]
Miyake and Martin [51]	Twitter, Facebook, blogs, news posts on social media, Reddit, forums, and other platforms	Public	January 1, 2020, to January 1, 2021	United Kingdom	English	No	Yes, but only of hashtags and emojis using IBM Watson emotional lexicon
Fu [34]	Twitter	Public	March 26, 2022, to April 26, 2022	United States	English	Yes, LDA [32]	Yes, VADER^c^ [38]
Ramakrishnan et al [52]	Twitter	Public	May 1 to Sep 30, 2021	Not specified	English	No	Yes, IBM Watson Tone Analyzer and 6 classical ML^d^ algorithms

^a^This table summarizes previous studies on topic modeling and sentiment analysis across various platforms and time frames. It includes information on the source of data, the population being studied, the time period covered, the geographic location, the language used, and the specific methodologies applied for topic modeling and sentiment analysis.

^b^LDA: latent Dirichlet allocation.

^c^VADER: Valence Aware Dictionary for Sentiment Reasoning.

^d^ML: Machine learning.

We have used new methods for our tasks that, according to our knowledge, have never been used before in the context of long COVID analysis. First, we used contextualized topic modeling (CTM) [53], which takes advantage of the entire context of the text rather than LDA, which, by applying neural variational techniques, uses a bag-of-words representation of the text, meaning that it ignores the order of words and considers only the frequency of words. Also, we use Llama 2 [54] to represent the text and classify its sentiment rather than rule-based methods or classical machine learning methods.

In summary, this paper addressed 3 key knowledge gaps related to long COVID and methods for topic modeling and sentiment analysis. First, it investigated topics and sentiments related to long COVID across 3 regions (Canada, the United States, and Europe), providing an opportunity for comparative analysis of issues and public perceptions in different geographical contexts. Second, the study introduced new techniques, such as CTM and large language model (LLM) sentiment analysis, to obtain nuanced insights related to long COVID. These advanced methods surpass traditional approaches by capturing the context and subtleties of the discourse more effectively. Third, the study integrated expertise from public health and computer sciences, combining methodological rigor with domain-specific knowledge to interpret the results in a meaningful way. This interdisciplinary approach ensures that the analytical methods are aligned with public health objectives and implications. By addressing these gaps, the study contributes valuable insights into public health monitoring and intervention strategies, demonstrating the use of social media analysis in understanding complex public health issues such as long COVID.

The innovation in this study lies in the integration of CTM and an LLM (Llama 2) for sentiment analysis. CTM goes beyond traditional LDA by capturing word meanings in context, leading to more coherent and semantically meaningful topics. Using Llama 2 for sentiment analysis provides a nuanced understanding of sentiments expressed in tweets, leveraging the model’s robust language comprehension capabilities.

## Methods

### Data and Data Processing

We used Twitter data for this analysis. In collaboration with public health experts working on long COVID, we identified relevant hashtags and keywords to extract Twitter data related to long COVID. Table 2 provides the list of hashtags and keywords that were used to collect the long COVID–relevant tweets.

**Table 2:** Terms used to collect long COVID–relevant tweets provided by health care professionals^a^.

Term	Description
#Long COVID	This hashtag refers to the long-term effects and symptoms experienced by individuals after recovering from COVID-19.
#mecfs	Abbreviation for “Myalgic Encephalomyelitis/Chronic Fatigue Syndrome,” a complex and debilitating condition characterized by extreme fatigue that is not alleviated by rest.
#MyalgicEncephalomyelitis	Also known as ME^b^, this is a chronic condition characterized by profound fatigue, pain, sleep disturbances, and other symptoms.
#Fibromyalgia	A chronic disorder characterized by widespread musculoskeletal pain, fatigue, and tenderness in localized areas.
#PostViralSyndrome	A condition that occurs after a viral infection, characterized by persistent symptoms such as fatigue, joint pain, and cognitive issues.
#Dysautonomia	A disorder of the autonomic nervous system, which controls involuntary bodily functions such as heart rate, blood pressure, and digestion. Symptoms can include lightheadedness, fainting, and difficulty regulating body temperature.
#ChronicFatigueSyndrome	Also known as CFS^c^, this is a complex disorder characterized by extreme fatigue that does not improve with rest and may be worsened by physical or mental activity.
#PwME	Abbreviation for “People with Myalgic Encephalomyelitis,” a term used to refer to individuals living with ME/CFS.
#MyalgicE	An abbreviation for ME, a chronic condition characterized by extreme fatigue and other symptoms.
#PostCovidSyndrome	A term used to describe the ongoing symptoms and health issues experienced by individuals after recovering from COVID-19.
#postcovid	A term used to describe symptoms or conditions that persist after recovering from COVID-19.
#PostViralFatigueSyndrome	A condition characterized by persistent fatigue and other symptoms following a viral infection.
#postviralillness	A term used to describe the lingering effects of a viral illness, which can include fatigue, muscle pain, and cognitive difficulties.
“Long Covid,” “long COVID,” and “long COVID syndrome”	A term used to describe the long-term effects of COVID-19, including persistent symptoms such as fatigue, shortness of breath, and cognitive issues.
“Post acute COVID”	A term used to describe the period of time after a COVID-19 infection where symptoms continue to persist.
“Post-COVID syndrome”	A term used to describe the syndrome of lingering symptoms and health issues experienced by individuals after recovering from COVID-19.
“Post-acute sequelae of SARS-CoV-2”	A term used to describe the ongoing symptoms and health issues experienced by individuals after recovering from COVID-19.
“Long-term COVID”	A term used to describe the ongoing symptoms and health issues experienced by individuals after recovering from COVID-19.
“Long haulers”	A colloquial term used to describe individuals who continue to experience symptoms and health issues after recovering from COVID-19.
“Chronic COVID syndrome”	A term used to describe the ongoing symptoms and health issues experienced by individuals after recovering from COVID-19.

^a^This table lists the hashtags and keywords identified by public health experts to extract tweets related to long COVID from Twitter. Each term includes a description to explain its relevance to long COVID.

^b^ME: myalgic encephalomyelitis.

^c^CFS: chronic fatigue syndrome.

We focused on English-language tweets due to the availability and accessibility of large volumes of Twitter data in English, allowing for more accurate and reliable analysis. Moreover, this focus enables a comparative study across regions where English is the predominant language, reducing the complexity of multilingual analysis and ensuring the consistency of sentiment and topic modeling methods.

The hashtags in Table 2 were selected based on their relevance to long COVID as identified by public health experts. These experts provided terms frequently used in discussions related to long COVID, ensuring comprehensive data collection. However, we acknowledge that some terms such as #Fibromyalgia, #Dysautonomia, and #ChronicFatigueSyndrome may not exclusively refer to long COVID. To mitigate this, we applied additional filtering and manual inspection to confirm that the majority of tweets were indeed related to long COVID. Despite these efforts, we recognize that some tweets may not be entirely relevant, and this limitation is addressed in the *Discussion* section.

For this study, we collected tweets alongside their metadata for the year 2021. We used the Twitter application programming interface [55] to extract the data, and afterward, we applied necessary preprocessing of the tweets. For preprocessing, we used the *tweet-preprocessor* toolkit, which applies cleaning, tokenizing, and parsing of URLs, hashtags, mentions, reserved words (“RT,” which stands for “Retweet” and “FAV,” which stands for “Favorite”), emojis, and smileys.

Among the 814,951 tweets extracted in total (Canada: n=98,796, United States: n=289,856, and Europe: n=426,299), we included only those tweets written in English using tweet metadata and the *spacy-langdetect* toolkit [56]. This process resulted in 782,089 tweets in total: 95,743 for Canada, 278,392 for the United States, and 407,954 for Europe including the United Kingdom. To remove tweet-specific keywords and URLs, we used the *tweet-preprocessor* toolkit [57]. We did not remove hashtags and mentions because they can be informative for our study. We lowercased and tokenized using the *Spacy* toolkit [58]. Since the methods we used in this paper are all unsupervised, we did not split the data for training and testing (we will share our data according to Twitter policy once this paper has been accepted).

### Topic Modeling

In order to analyze the public perception of long COVID, we applied CTM. CTM uses pretrained representations of language (eg, bidirectional encoder representations from transformers) to support topic modeling based on the context. To identify the best number of topics that can be extracted from the tweets with the best coherence and diversity between the topics, we applied the following automatic evaluation metrics with different settings: for coherence, we used normalized pointwise mutual information [59], Cv [60], and word embedding [61], while for diversity, we used topic diversity and inverted rank-biased overlap [62]. These metrics evaluate the semantic consistency and distinctiveness of topics, ensuring meaningful results.

First, we applied the measures on 5, 10, 15, and 20 topics. However, the best topic coherence and diversity metrics results were obtained by extracting 5 topics. We tried the same metrics over 4 and 6 topics to see whether they would have better results, but they did not. Moreover, to confirm the automatic evaluation we have shown the 5 extracted topics to the health care professionals.

To assess changes in topics of discussion over time, we compared timelines of topic distributions across the 3 regions and examined how this reflected change in public perception of long COVID. To discover topics and track the topic change over time, we constructed topic models on our Twitter data using zero-shot CTM implementation.

We used the *pyLDAvis* tool [63] to visualize the 5 topics generated by the CTM model. *PyLDAvis* visualizes the relationship between topics using multidimensional scaling. It helps understand topic overlap and distinctiveness, aiding in the interpretation of topic coherence and diversity metrics. This methodological detail enhances the reliability of our topic modeling results. Two public health experts examined the visualization and one of them labeled each topic to make it easier to understand the context of each topic. More specifically, we performed a basic analysis based on an examination of the estimates of the vector, a document-to-topic distribution, produced by the model. We first divided tweets into weekly buckets using Coordinated Universal Time–12 time stamps (eg, January 21-26, January 27 to February 2, and February 3-9, 2021). We then computed a mean vector for tweets in each bucket as done by Griffiths and Steyvers [64].

### Sentiment Analysis

Sentiment analysis was conducted to gauge the emotional tone expressed in tweets related to long COVID. The objective was to understand how individuals on Twitter perceived and communicated their sentiments regarding long COVID throughout the year 2021. To analyze the sentiments expressed in tweets related to long COVID, we used sentiment analysis at the tweet level. For tweet sentiment classification, we used Llama 2, which have been recognized as one of the most widely used LLMs recently. The robustness of Llama 2 for sentiment analysis is supported by recent studies demonstrating its effectiveness across various domains [65-68].

The accuracy of sentiment analysis using Llama 2 was validated through manual inspection of a random sample of classified tweets. Misclassification rates were low, demonstrating the model’s robustness. Prompts were designed to capture the overall sentiment of each tweet accurately, considering context and phrasing nuances.

The sentiment analysis pipeline provided by Hugging Face’s Transformers library is designed to analyze the sentiment of a given piece of text. Pipelines are high-level objects that allow users to easily apply pretrained models to various natural language processing (NLP) tasks. This specific pipeline uses the Llama 2 model for sentiment analysis, facilitating efficient processing of text data. Through tokenization, encoding, and model inference, the pipeline predicts sentiment, categorizing it as positive, negative, or neutral. This process offers valuable insights into the emotional tone of tweets related to long COVID.

### Ethical Considerations

This study was conducted using publicly available Twitter data, which are anonymized and deidentified. The data collection and analysis were carried out in accordance with Twitter’s terms of service. No personal or sensitive information was accessed, ensuring the privacy and confidentiality of Twitter users.

As the research involved publicly available, anonymized data from Twitter, it did not require formal ethics review approval. This approach is consistent with TCPS 2 (Tri-Council Policy Statement: Ethical Conduct for Research Involving Humans), article 2.2, regarding secondary analysis of public data where no direct interaction with human subjects occurs.

Given that only publicly accessible data were analyzed, specific informed consent from Twitter users was not required. The original consent provided by Twitter users for public sharing of their data under Twitter’s terms of service allows for secondary analysis without additional consent.

All data analyzed in this study were anonymized and deidentified. No identifying information was collected, and data were handled in ways that fully comply with ethical guidelines on protecting participant confidentiality.

No compensation was provided to Twitter users, as the study used only publicly available data and did not involve direct participation or interaction with users.

## Results

### Topic Modeling

The 5 topics identified through topic modeling and a domain expert effort were as follows:

1. Long COVID in people including children in the context of vaccination (T1)
2. Duration and suffering associated with long COVID (T2)
3. Persistent symptoms of long COVID (T3)
4. The need for research on long COVID treatment (T4)
5. Measuring long COVID symptoms (T5)

A set of sample tweets associated with each topic was then examined to qualitatively validate the topic labels. The sample tweets provided contextual information that could not be inferred from the salient terms alone. Table 3 highlights the topics and their labels.

**Table 3:** Labels for top 5 prevalent topics in Canada, the United States, and Europe relating to long COVID^a^.

Topic number	Salient terms (top 10 in italics)	Label based on salient terms	Label based on sample tweets
T1	“*people,” “children,” “kids,” “covid,” “long,” “get,” “many,” “risk,” “vaccinated,” “deaths,”* “know,” “cases,” “vaccine,” “schools,” “death,” “even,” “also,” “still,” “die,” “amp,” “term,” “vaccines,” “young,” “school,” “delta,” “health,” “protect,” “masks,” “spread,” and “immunity”	Long COVID in people, including children, in the context of vaccination	More objective perspectives: research, monitoring, experience, and news items highlighting significant impact of symptoms of long COVID in adults and children, including potential benefits of vaccination in reducing long COVID risk
T2	“*day,” “feel,” “like,” “back,” “days,” “year,” “got,” “time,” “pain,” “last,”* “week,” “feeling,” “months,” “since,” “one,” “better,” “friend,” “ago,” “still,” “felt,” “work,” “went,” “hope,” “years,” “body,” “go,” “march,” “going,” “first,” and “today”	Duration and suffering associated with long COVID	More subjective perspectives: suffering and frustration associated with long COVID
T3	“*symptoms,” “19,” “covid,” “long,” “study,” “post,” “patients,” “term,” “infection,” “haulers,”* “new,” “covid19,” “months,” “syndrome,” “coronavirus,” “fatigue,” “acute,” “effects,” “common,” “studies,” “viral,” “via,” “persistent,” “reported,” “sars,” “found,” “weeks,” “experiencing,” “brain,” and “lingering”	Persistent symptoms of long COVID	Persistent symptoms of long COVID, with greater focus on formal and informal advocacy and awareness-raising around long COVID
T4	“*longcovid,” “mecfs,” “research,” “please,” “cfs,” “support,” “amp,” “pwme,” “help,” “thank,”* “patients,” “community,” “need,” “share,” “us,” “longcovidkids,” “funding,” “sign,” “patient,” “awareness,” “join,” “millionsmissing,” “myalgicencephalomyelitis,” “treatments,” “illness,” “treatment,” “guidelines,” “nicecomms,” “needs,” and “thanks”	Need for research on long COVID treatment	Calls to address the plight of people with long COVID, either by researchers or by governments, or frustration at having been neglected, and frustration at having ongoing symptoms.
T5	“*fewer,” “ontario,” “vast,” “matters,” “measure,” “amongst,” “likelihood,” “possibility,” “polio,” “contract,”* “ha,” “tracking,” “victims,” “de,” “clinically,” “eg,” “vs,” “admitted,” “telegraph,” “exposure,” “estimate,” “fda,” “surge,” “impacted,” “reducing,” “70,” “american,” “ages,” “yo,” and “largely”	Measuring long COVID symptoms	The large number of people affected by, or projected to be affected, by long COVID symptoms, and associated societal impacts.

^a^Derived from topic modeling of Twitter content from 2021, with labels for each topic based on human interpretation of keywords and associated sample tweets. This table shows the topics identified, the salient terms associated with each topic, and the final labels given to each topic based on the interpretation of sample tweets.

Through expert’s evaluation, it became clear that the accurate and meaningful labels could not be consistently developed through salient terms alone, devoid of the context of the sample tweets from which they were derived. In particular, we observed that topic T1 tended to be associated with more objective perspectives on long COVID such as research, monitoring, objective descriptions of experiences, and news items, whereas T2 tended to be associated with more subjective perspectives such as suffering and frustration associated with having long COVID. In addition, the sample tweets suggested that T3 and T4 had advocacy for more to be done to address long COVID as a prominent element. Finally, T5 had societal impacts as a prominent element within the sample tweets.

**Figure 1:**
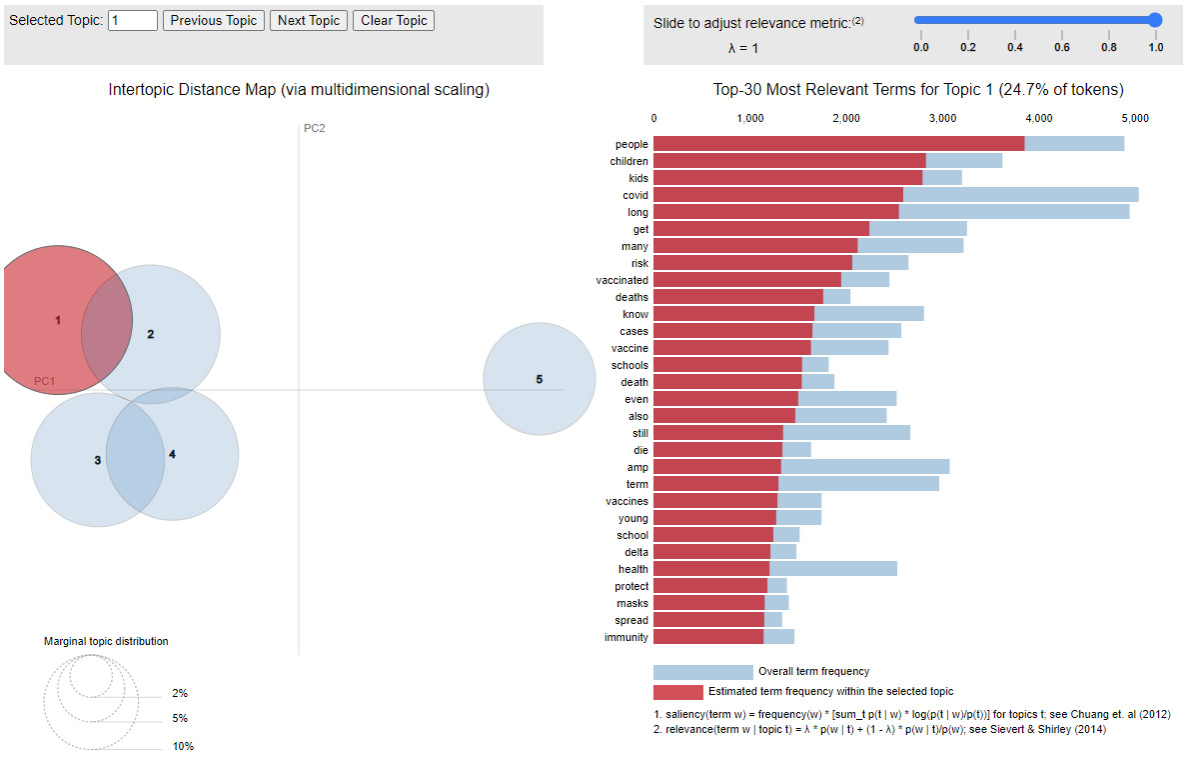
Intertopic distance map via multidimensional scaling. This figure visualizes the distance between the 5 topics identified from long COVID–related tweets in 2021, showing intertopic overlap among topics T1-T4 but not for T5. The study includes tweets from Canada, the United States, and Europe. T1: Long COVID in people including children in the context of vaccination; T2: duration and suffering associated with long COVID; T3: persistent symptoms of long COVID; T4: need for research on long COVID treatment; and T5: measuring long COVID symptoms. In the figure, T1 is selected.

Examination of sample tweets by the health care professionals also revealed overlap in the content between some of the topics. This is also reflected to some extent in the intertopic distances that were computed and visualized in Figure 1, which demonstrates some intertopic overlap among topics T1-T4 but not for T5. Since *pyLDAvis* is an interactive visualization of the topics, we asked the health care professionals to explore the adjacent metric settings. By default, *pyLDAvis* is set for λ=1, which sorts words just by their frequency within the specific topic (by their red bars). By contrast, setting λ=0 words sorts words by their “lift.” This means that words whose red bars are nearly as long as their blue bars will be sorted at the top.

The types of discrepancies found between the salient terms–based topic labels and some of the sample tweets were due to low-label specificity, misrepresentation, or topic overlap. Some tweets were also found to be irrelevant to long COVID altogether; in particular, a portion of the sample tweets was irrelevant because of misclassification of long COVID–related tweets with tweets on COVID-19. Examples of these discrepancies are shown in Multimedia Appendix 1.

**Figure 2:**
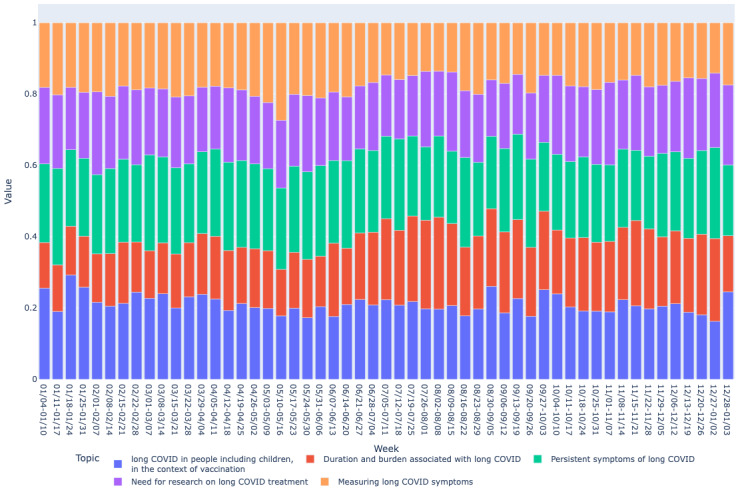
Analyzing topic trends: visualizing the prominence of different topics over time through a bar chart for the Canada region. This figure shows the weekly distribution of the 5 identified topics in long COVID–related tweets in Canada during 2021.

**Figure 3:**
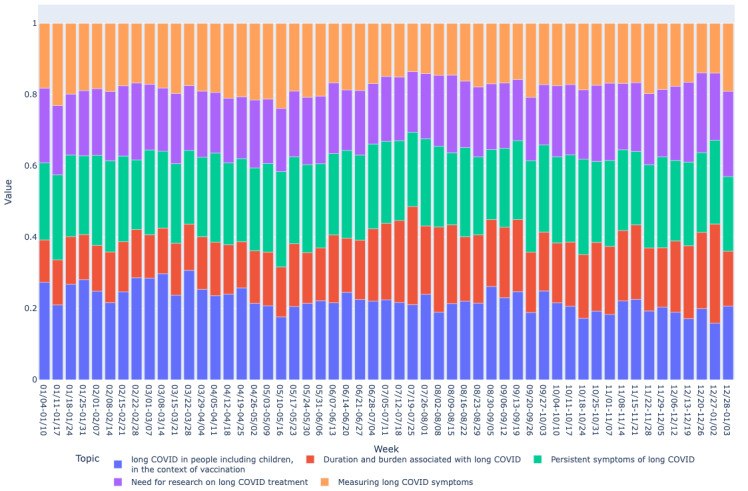
Analyzing topic trends: visualizing the prominence of different topics over time through a bar chart for the US region. This figure displays the weekly distribution of the 5 identified topics in long COVID–related tweets in the United States during 2021.

**Figure 4:**
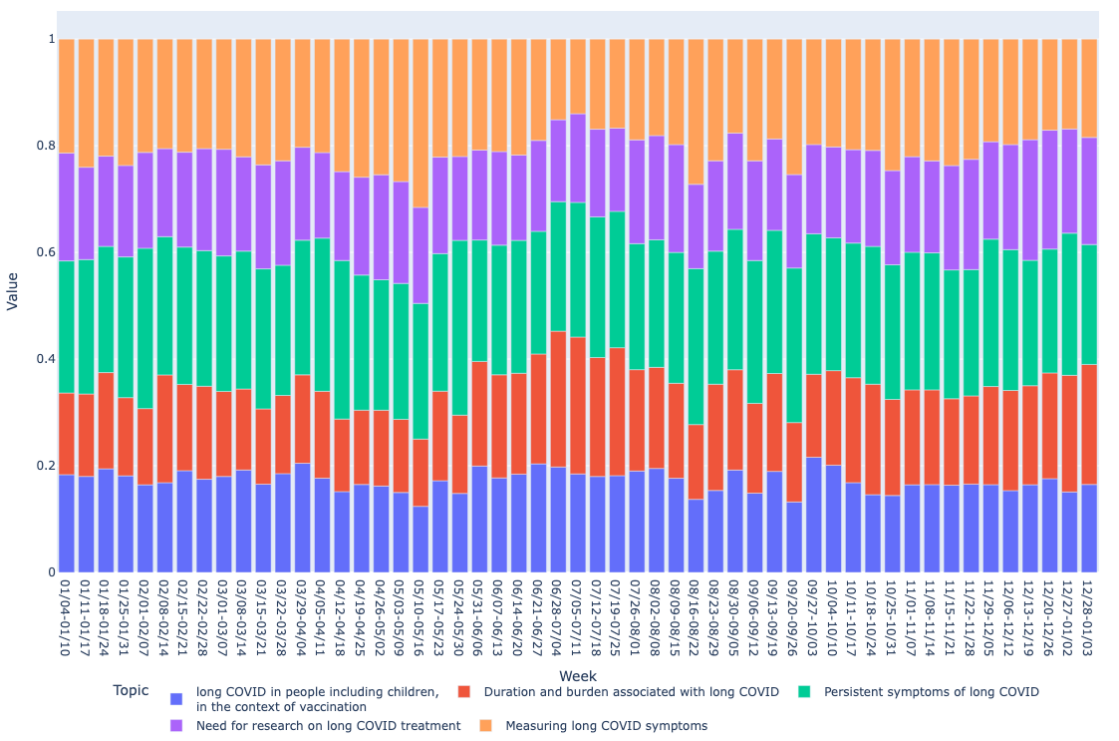
Analyzing topic trends: visualizing the prominence of different topics over time through a bar chart for Europe region. This figure depicts the weekly distribution of the 5 identified topics in long COVID–related tweets in Europe during 2021.

**Figure 5:**
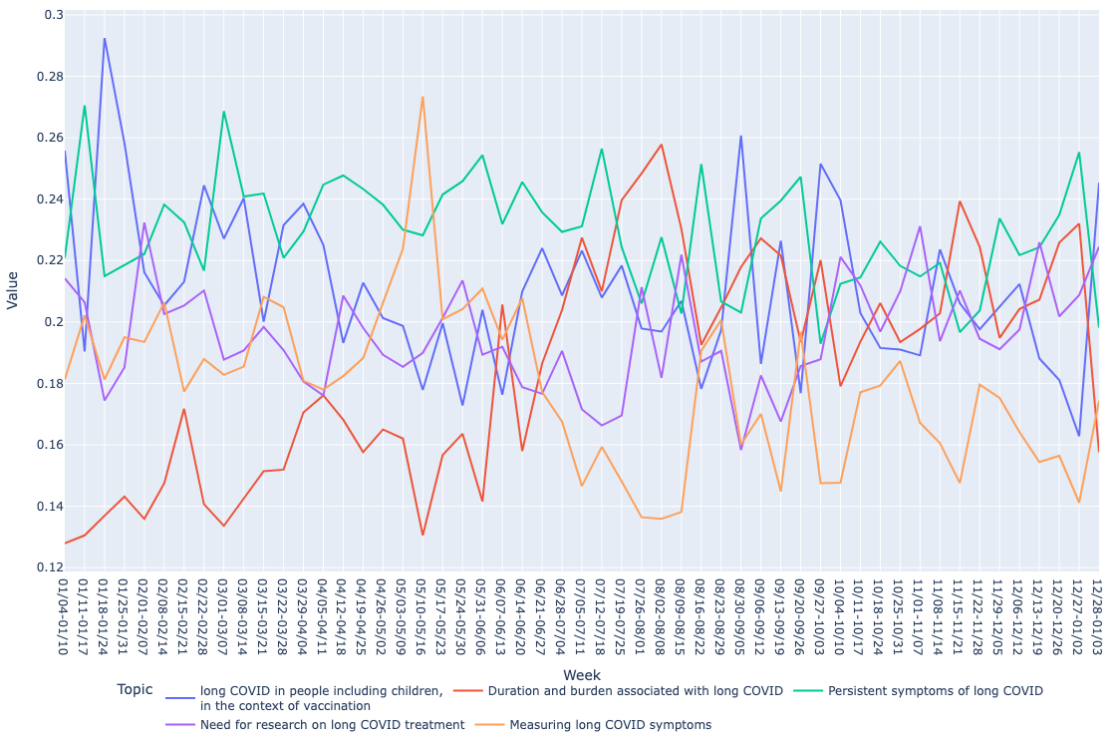
Analyzing topic trends: visualizing the prominence of different topics over time through a line chart for the Canada region. This figure shows the trends of the 5 identified topics in long COVID–related tweets in Canada over the year 2021.

**Figure 6:**
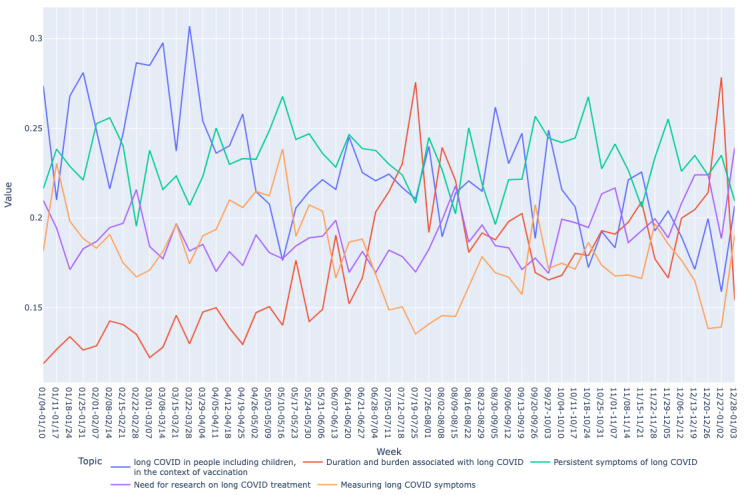
Analyzing topic trends: visualizing the prominence of different topics over time through a line chart for the US region. This figure illustrates the trends of the 5 identified topics in long COVID–related tweets in the United States over the year 2021.

**Figure 7:**
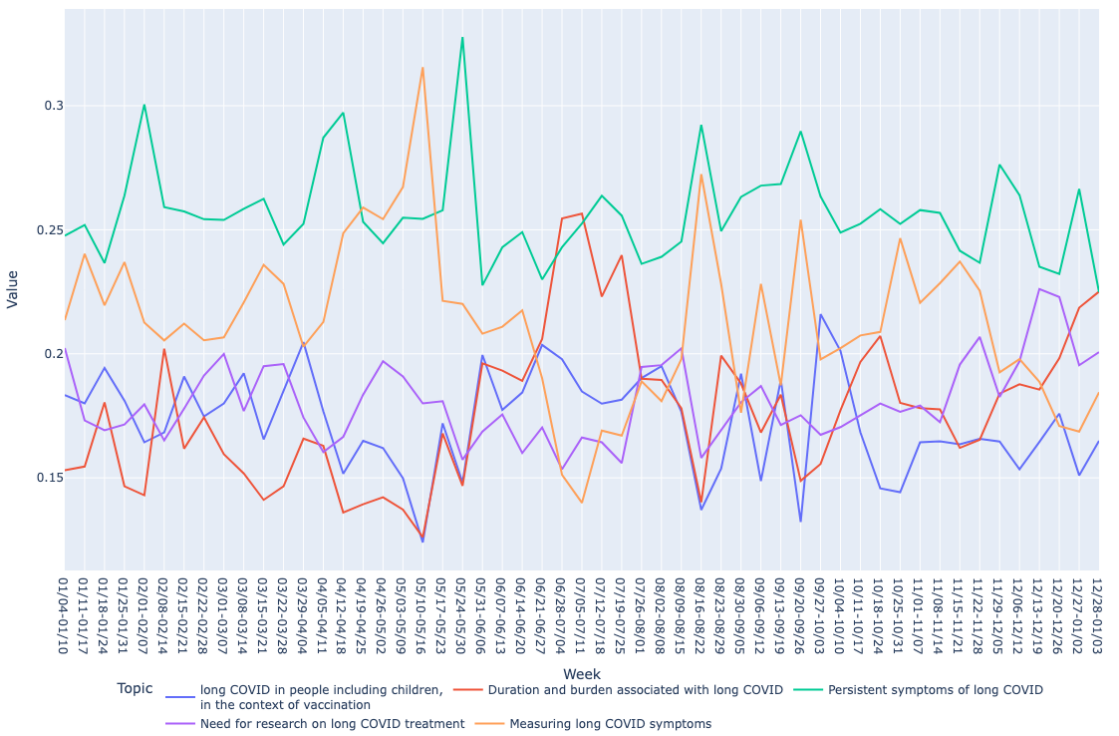
Analyzing topic trends: visualizing the prominence of different topics over time through a line chart for Europe region. This figure shows the trends of the 5 identified topics in long COVID–related tweets in Europe over the year 2021.

Based on the mean vector for each bucket, we drew graphs of long COVID topics over time as shown in Figures 2-7. We observed similar patterns between tweets in Canada and the United States. For example, the topic about subjective experiences around the duration and suffering associated with long COVID (T2) was less prominent early in the year but increased in prominence over time, peaking in late July-early August, and remained higher for the rest of the year, while there was no discernible trend in Europe. On the other hand, the topic on more objective perspectives on long COVID (T1) was relatively low in prominence in Europe compared with Canada and the United States. In contrast, the topic on persistent symptoms of long COVID (T3) was prominent throughout the year in all 3 regions.

Both bar and line graphs were used to provide different perspectives on the data. Bar graphs offer a snapshot of weekly topic distribution, while line graphs illustrate trends over time, providing a comprehensive understanding of topic dynamics.

### Sentiment Analysis

The sentiment distribution across long COVID–related tweets shows that there was no specific pattern. However, there was an increase in negative sentiment in July and August, followed by an increase in positive sentiment in September and again an increase in negative sentiment in December. In the United States, the trend was different, with a large amount of negative sentiment from January to March, a spike in July, and continued negative sentiment during August and September. April was notable for more positive sentiment and low negative sentiment. In particular, July had a lot of activity with some positive sentiment in addition to a large number of negative-sentiment tweets. The pattern in Europe was also different, with a large number of positive-sentiment tweets and substantial negative-sentiment tweets throughout the year.

There are many plausible explanations for these swings in positive and negative sentiments. For example, in July 2021, US President Joe Biden said that the long-term effects of COVID-19 can be considered a disability under federal civil rights laws, which may have spurred some positive sentiments [69]. In July, the National Institutes of Health also announced US $40 million for the study of long COVID and multisystem inflammatory syndrome in children [70]. In addition, multiple studies presented both positive and negative news about long COVID. All of these may have spurred both negative and positive discourse related to long COVID.

While we cannot establish a direct causal relationship, potential influences on sentiment swings can be hypothesized. Significant events, such as US President Joe Biden’s statement in July 2021 that the long-term effects of COVID-19 could be considered a disability under federal civil rights laws, and the National Institutes of Health’s announcement of US $40 million for the study of long COVID and multisystem inflammatory syndrome in children, likely contributed to these fluctuations. In addition, the presentation of both positive and negative news about long COVID in various studies may have further influenced public sentiment. These factors illustrate the complexity of inferring causality from observational data and highlight the multifaceted nature of public discourse.

**Figure 8:**
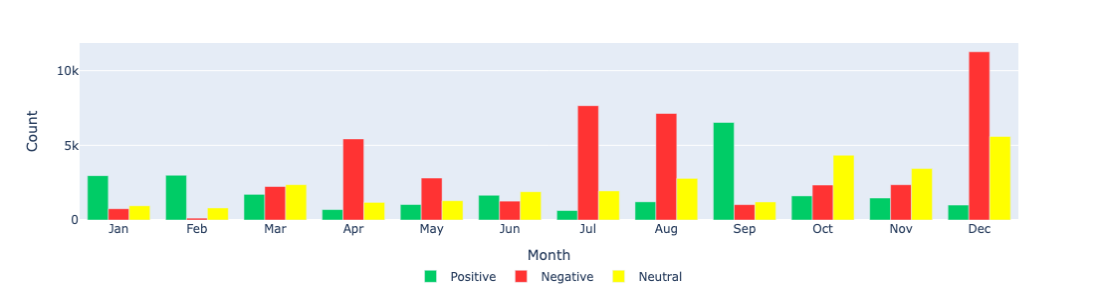
Sentiment analysis: visualizing the monthly sentiment counts produced by Llama 2 through a bar chart for the Canada region. This figure displays the count distribution of positive, negative, and neutral sentiments in long COVID–related tweets in Canada during each month of 2021.

**Figure 9:**
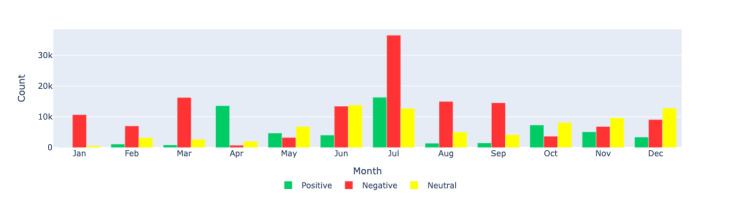
Sentiment analysis: visualizing the monthly sentiment counts produced by Llama 2 through a bar chart for the US region. This figure shows the count distribution of positive, negative, and neutral sentiments in long COVID–related tweets in the United States during each month of 2021.

**Figure 10:**
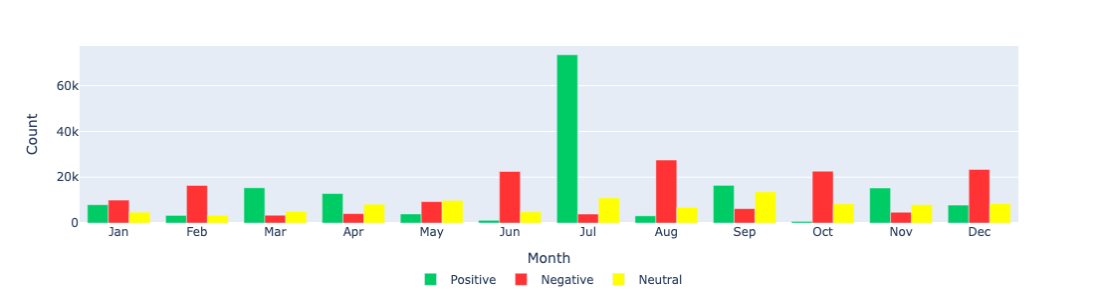
Sentiment analysis: visualizing the monthly sentiment counts produced by Llama 2 through a bar chart for Europe region. This figure illustrates the count distribution of positive, negative, and neutral sentiments in long COVID–related tweets in Europe during each month of 2021.

Figures 8-10 show the count distributions of positive, negative, and neutral sentiments for the Canada, US, and European regions, respectively, providing a detailed view of the monthly sentiment counts. These figures help identify specific periods of increased positive or negative sentiments, which can be correlated with significant events.

**Figure 11:**
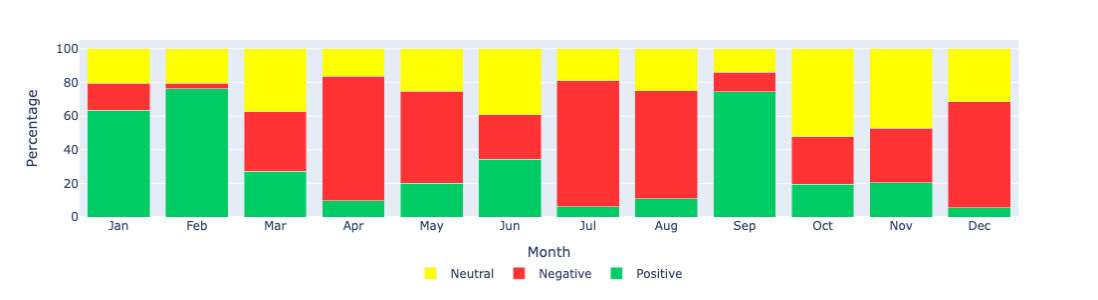
Sentiment analysis: visualizing the monthly sentiment percentages produced by Llama 2 through a bar chart for the Canada region. This figure displays the percentage distribution of positive, negative, and neutral sentiments in long COVID–related tweets in Canada during each month of 2021.

**Figure 12:**
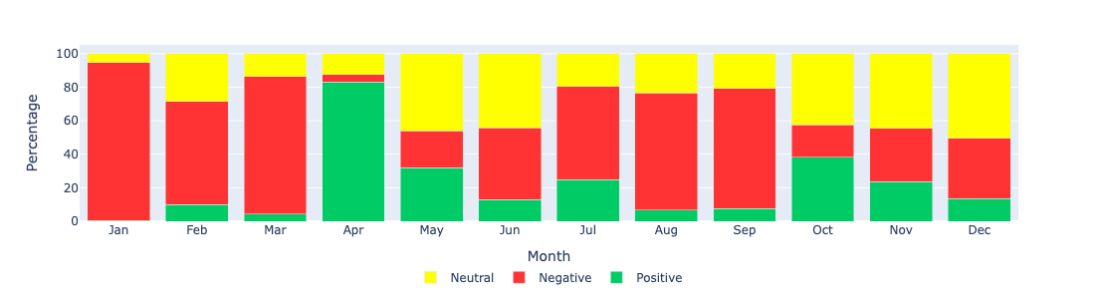
Sentiment analysis: visualizing the monthly sentiment percentages produced by Llama 2 through a bar chart for the US region. This figure shows the percentage distribution of positive, negative, and neutral sentiments in long COVID–related tweets in the United States during each month of 2021.

**Figure 13:**
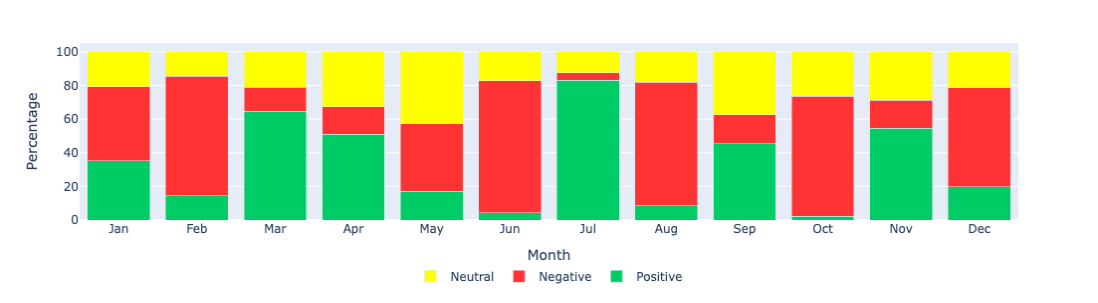
Sentiment analysis: visualizing the monthly sentiment percentages produced by Llama 2 through a bar chart for Europe region. This figure illustrates the percentage distribution of positive, negative, and neutral sentiments in long COVID–related tweets in Europe during each month of 2021.

Figures 11-13 show the percentage distributions of positive, negative, and neutral sentiments for the same regions, providing additional insights into the proportionate sentiment trends over the months. These figures complement the count-based visualizations by showing the relative proportions of each sentiment category, offering a more nuanced understanding of the sentiment landscape.

## Discussion

### Principal Findings

In this study, we used topic modeling and sentiment analysis on Twitter data from Canada, the United States, and Europe in 2021. We identified 5 key topics: objective and subjective descriptions of the experience of people living with long COVID; persistent symptoms associated with long COVID; the need for more attention to be given to long COVID by researchers and governments; and measuring the impact of long COVID. The overlapping nature of these topics is likely due to the complex and ill-defined nature of long COVID, which shares symptoms with other conditions. Sentiment analysis showed a mix of positive and negative sentiments over time, with notable variations across different countries. The fluctuations in sentiment may be influenced by various events and news during the study period. For instance, misinformation such as claims that long COVID is merely psychological and not a real medical condition could lead to negative sentiments and confusion among the public. These insights into discussions and sentiments related to long COVID can help understand patient concerns and combat misinformation. The code used for data analysis is available in the study by AbuRaed [71].

The combined use of topic modeling and sentiment analysis in this study provided a richer, multidimensional view of the public discourse on long COVID. Topic modeling identified key themes such as persistent symptoms (T3) and the need for more research on treatments (T4), while sentiment analysis added an emotional layer to these discussions. For example, negative sentiments were consistently associated with topics about suffering and the call for more research, reflecting public frustration with the ongoing impact of long COVID. In contrast, topics such as vaccination (T1) often displayed more balanced or positive sentiments, indicating public optimism during vaccine rollout periods. By integrating these 2 approaches, we captured not only what people were discussing but also how they felt about these issues, offering a more comprehensive understanding of public sentiment and its fluctuations over time. This insight is crucial for public health communication and intervention strategies, as it highlights the emotional responses tied to different aspects of the long COVID discourse.

Most previous studies focused on either topic modeling or sentiment analysis related to long COVID but not both. In addition, many of these studies used LDA for topic modeling, which operates under the bag-of-words assumption and ignores word order. We used CTM, which captures word meanings in context, allowing for more coherent and semantically meaningful topics. For sentiment analysis, we used Llama 2, instead of rule-based or hybrid systems, demonstrating its robustness in sentiment classification. Our findings showed both consistencies and discrepancies compared with existing studies, underscoring the robustness of our identified topics and sentiment trends while also highlighting the nuanced capabilities of our methodology.

CTM addresses the limitations of traditional methods by capturing word meanings in context, enhancing topic coherence and relevance. This approach is particularly suited for complex and nuanced topics such as long COVID.

The topic labels, based on salient terms, were found to be inadequate upon examination of sample tweets. This discrepancy is expected due to the nonrepresentative nature of sample tweets and the overlapping representation among topics T1-T4, as indicated by the intertopic distances. Misinterpretation due to word-sense disambiguation challenges also contributed to misrepresentative labeling. Salient terms such as “like” or “long” are difficult to interpret without context, leading to irrelevance in the labels concerning long COVID.

The evaluation of topic labels involved expert review of sample tweets, ensuring that labels accurately reflected tweet content. This iterative process improves label accuracy and relevance. The methodology and results of this evaluation are detailed in the “Methods” and “Results” sections.

When examining topic prominence over time, it was challenging to identify clear patterns due to the overlapping nature of topics. However, there was an overall increasing trend in discussions around the duration and suffering associated with long COVID, consistent with the growing awareness and burden of long COVID as the pandemic progressed.

### Public Health Implications

The results of this study have significant public health implications. Monitoring discussions and perceptions of people with long COVID can provide valuable insights into their needs and changing issues over time. Although geographical analysis is limited due to data constraints, this system can assess the impact of various actions and measures across jurisdictions. Furthermore, this approach can be applied to other emerging public health issues to monitor discourse, address concerns, and correct misinformation.

By identifying key topics and sentiment trends, our study can inform public health messaging and interventions to address misinformation. For example, if we observe a spike in negative sentiment and misinformation regarding the efficacy of long COVID treatments, public health officials can launch targeted information campaigns to provide accurate information and resources about available treatments and their effectiveness. Understanding public concerns and sentiment shifts allows for these targeted information campaigns to correct false narratives and provide timely support to affected individuals.

### Limitations

There were several limitations to this study related to the data source, data collection and processing, the NLP methods used, and the approach to interpretation of the outputs.

With regard to the data source, a portion of the tweets were cut off, either because Twitter’s character limit per post forced the user to post multiple tweets to express their thoughts or because of the extraction process itself. In addition, some tweets are repeated multiple times (sometimes more than 80 times), because of retweets. Moreover, because of the design of the Twitter platform, such as character limits, and the intentions of Twitter users (eg, to grab people’s attention), the ideas expressed are abbreviated and unclear. We extracted only those tweets that were in the English language and further limited tweets to those originating from Canada, the United States, and Europe. Thus, the data are unlikely to be representative of discussions and perceptions on long COVID elsewhere in the world. Finally, Twitter users constitute a small subset of the world population, further limiting the representativeness of these data.

With regard to data collection and data processing, the collection of Twitter data related to long COVID using long COVID–related keywords was subject to misclassification, since tweets relating to long COVID had significant overlap with tweets unrelated to long COVID. While this misclassification cannot be quantified without examining and categorizing each tweet one by one, an examination of a random sample of 164 tweets identified that approximately 13% (21/164) of the tweets were unrelated to long COVID. This misclassification may be in part because of the still poorly defined nature of long COVID as a condition, and in part a result of the long COVID–related keywords that were used to extract the tweets. For example, fibromyalgia, a chronic condition with similar symptomatology to long COVID, was discussed in some tweets that were irrelevant to long COVID.

With respect to NLP methods used to analyze the Twitter data, there was significant overlap between many of the topics in the topic model, as discussed above. In addition, challenges with word-sense disambiguation limit the interpretability of results.

With respect to the approach to interpretation of the outputs, having 1 domain expert to label the topics based on the sample tweets and a second domain expert to label the topics based on the salient terms would have helped enhance objectiveness in the labeling process. In this study, since the same domain expert did the labeling using these 2 approaches, there was likely some bias in how the labels were determined. Moreover, while no formal method was applied in this study to account for how public health measures impacted public perceptions on long COVID, the change in public health measures over time and region over the course of 2021 does constitute a potential source of bias and confounding in our analysis. For example, more stringent measures may prompt a more favorable perception toward government among those concerned about long COVID, since stringent measures may be perceived as part of an effort to reduce the risk to individuals not only of COVID-19 but also of long COVID. Conversely, less stringent measures may be perceived less favorably for similar reasons.

### Conclusions

Our key findings include the identification of 5 main topics related to long COVID, significant regional variations in public discourse, and the fluctuating nature of sentiments influenced by major events. These findings provide a nuanced understanding of public concerns and the potential to inform public health strategies.

This study shows exploratory results from topic modeling and sentiment analysis on long COVID–related tweets in Canada, the United States, and Europe. By comparing results across these regions, we demonstrated changes in topic prominence over time. Public health domain experts played a crucial role in interpreting the results, emphasizing the importance of a human-in-the-loop approach in sentiment analysis. Although we identified some regional and temporal differences in topics, the main interpretation is the evolving public discourse and increasing awareness of long COVID throughout 2021.

The insights gained from this study can help public health officials and policy makers design more effective communication strategies and interventions tailored to the needs and concerns of those experiencing long COVID. By understanding the evolving public sentiment and key issues discussed on social media, public health responses can be better aligned with the population’s needs. This research contributes to the broader understanding of how social media data can be leveraged for public health surveillance and intervention.

### Future Directions

Future studies should explore more refined methods to enhance the specificity and accuracy of topic modeling and sentiment analysis. In addition, integrating more diverse social media platforms and languages could provide a more comprehensive understanding of global long COVID discourse. Finally, continuous monitoring and real-time analysis of social media data can further aid in understanding public perceptions and developing timely public health interventions.

## Data Availability

The datasets generated and analyzed during this study will be made available in the Zenodo repository [71] after the acceptance of the paper. The code used for data analysis is available as mentioned in the *Discussion* section. To comply with Twitter’s terms of service, shareable data include aggregated results, anonymized data, and Tweet IDs, whereas nonshareable data include raw tweets, user profile information, and any nonpublic data.

## Appendix

Multimedia Appendix 1 Sample tweets.

## Abbreviations

CTM: contextualized topic modeling
LDA: latent Dirichlet allocation
LLM: large language model
NLP: natural language processing
