# Supplementary material for: Long COVID Discourse in Canada, the United States, and Europe: Topic Modeling and Sentiment Analysis of Twitter Data
Source: J Med Internet Res. 2024 Dec 9;26:e59425. doi: 10.2196/59425 (PMC11667136; doi:10.2196/59425)
Supplement: Multimedia Appendix 1 [file jmir_v26i1e59425_app1.docx]

Multimedia Appendix 1. Sample tweets demonstrating low specificity, misrepresentation, and overlapping representation of the salient terms–based topic labels, and irrelevance with respect to Long COVID.^a^

| Low specificity  ·   T1: “When health systems announce downstream hospital'n and ICU beds, focus on these metrics - we ignore the **long**-COVID and socioecon implications at individual and macro levels. We ignore that &gt;95% of COVID is managed by PCP in community (not on the first lists for **vaccination**)”—*The T1 label is not specific with respect to what the context of vaccination is for the tweets; this tweet demonstrates one specific context in which vaccination is discussed.*  Misrepresentation  ·   T2: “@andrewkujavsky People need to understand why they should be tested: 1. Know how long to quarantine. 2. Know how to protect others. 3. Quicker treatment if things go bad. 4. Know whether or not to get the vaccine when your **time** comes. 5. If #LongCovid, have medical system treat you seriously.”—*This tweet refers to “time” not in the context of Long COVID, inconsistent with the T2 label “duration and suffering associated with Long COVID”.*  ·   T2: “#variantwave. #zeroCovidCanada. NOW. It's not a matter of ideology. Just cold hard facts. No ethical wiggle room **like** **last** year. Anything but elimination action in face of this is - absolutely no question - conscious decision to NOT protect citizens from #LongCovid &amp; more deaths.”—*This tweet does not discuss “duration and suffering associated with Long COVID”.*  Topic overlap  ·   T2: “@aliciamcauley I’d also point out that many people who survive, esp. long-haulers and people hospitalized/on ventilators, often have long-term health effects, **like** breathing issues, heart problems, brain fog, poor circulation &amp; more. Wellness &amp; quality of life of survivors is another big issue.”—*This tweet’s content overlaps with the T3 label “Persistent symptoms of Long COVID”.*  Irrelevance with respect to Long COVID  ·   T1: “Experts say there is not enough data yet to know how **Long COVID**-19 **vaccines** provide immunity for”—*This tweet does not discuss Long COVID, but rather the duration of immunity for the vaccine.*  ·   T1: “Seniors suffering from total isolation in B.C. care homes with months-**Long COVID**-19 outbreaks, families say”—*This tweet does not discuss Long COVID, but rather the duration of COVID-19 outbreaks in long-term care homes.*  ·   T3: “Don't Just Recover From The Pandemic—Emerge Stronger With These 4 Technologies #technology #businessowner #postCOVID”—*This tweet does not discuss Long COVID, but rather the recovery from the pandemic.*  ·   T4: “#Fibromyalgia "...quick fix…it’s just that I never expected one. And seventeen years later, with still no quick fix in sight, I’m pretty thankful I didn’t count on one." #WhatIKnowIsRea”—*This tweet does not discuss Long COVID, but rather fibromyalgia, a condition for which some parallels have been drawn with Long COVID.* |
| --- |

^a^Salient terms are in bold font.
